# Characterization and management of cystic pancreatic neoplasms communicating lesions (IPMN)

**DOI:** 10.1186/1470-7330-14-S1-O26

**Published:** 2014-10-09

**Authors:** Giovanni Morana, Matteo Gazzola, Alex Faccinetto, Giancarlo Addonisio, Alberto Dorigo

**Affiliations:** 1 Radiological Department, General Hospital Ca’ Foncello Treviso (IT), Italy; 2 Radiological Department, University of Padova, Italy

## Introduction

Intraductal papillary mucinous neoplasms (IPMNs) are a group of exocrine mucin-producing tumors, diagnosed at a mean age of 60 years, with a male prevalence [1].

Improvements in imaging techniques have led to an increasing incidental detection of IPMNs: the prevalence of incidental cystic pancreatic lesions can be observed in up to 19.6% of imaging studies [2].

Three types of IPMNs have been described [1]: the main duct type; the branch-duct type and the mixed type, which meet the criteria for both MD-IPMN and BD-IPMN, with significant differences in frequencies of malignancy in IPMNs according to the morphological types, higher for MD-type (mean 61.6%) and lower for BD-type (25.5%)(3).

## Imaging-pathologic correlations

### Pathologic features

IPMNs appear with a cystic dilation of the involved segment, either main duct and branch duct. Some findings can be suggestive of the behavior of the IPMN, according to the presence of high risk stigmata or worrisome features [3].

High risk stigmata suggest the high possibility that the lesion is malignant, thus requiring resection if the patient is surgically fit: main duct diameter > 10 mm for MD-IPMN, the presence of solid enhancing nodules within the cyst in BD-IPMN, or obstructive jaundice in presence of a cystic lesion of the pancreatic head.

Worrisome features suggest the possibility that the lesion could evolve as malignant, thus requiring further workup by EUS, to better risk-stratify the lesion, and a strict follow-up: cyst > 3 cm, thickened enhanced cyst walls, MPD size of 5-9 mm, non-enhancing mural nodules, abrupt change in the MPD caliber with distal pancreatic atrophy, and lymphadenopathy.

### Imaging features

MR with MRCP has the highest capacity to assess the presence of communication with main pancreatic duct, with a sensitivity of 91.4-100% [4].

The proliferating nodule is characterized by the capacity to enhance after contrast media administration, which can be appreciated with all imaging technique (CEUS; CT; MRI), after administration of contrast media.

In case of IPMN, MDCT has a sensitivity of 70% in the diagnosis of benignity vs malignancy according to some worrisome features (nodules, main pancreatic duct > 10 mm, thick septa, calcifications) [5].

MR with MRCP has a sensitivity, specificity and accuracy of 70%, 92% and 80%, respectively in the diagnosis of benignity vs malignancy according to some worrisome features (nodules, main pancreatic duct > 10 mm, thick septa, calcifications)[5].

## Management

International consensus guidelines [3] recommend resection in presence of high-risk stigmata, while in presence of “worrisome features” the lesion should be evaluated by EUS to further risk-stratify the lesion. Age, status of the patient can have influence on the decision management.

## Conclusions

Age, clinical, laboratory and imaging findings are accurate in stratifying these lesions, and imaging plays a pivotal role in their management.
